# New Insights of the NEET Protein CISD2 Reveals Distinct Features Compared to Its Close Mitochondrial Homolog mitoNEET

**DOI:** 10.3390/biomedicines9040384

**Published:** 2021-04-05

**Authors:** Myriam Salameh, Sylvie Riquier, Olivier Guittet, Meng-Er Huang, Laurence Vernis, Michel Lepoivre, Marie-Pierre Golinelli-Cohen

**Affiliations:** CNRS, Institut de Chimie des Substances Naturelles, Université Paris-Saclay, UPR 2301, 91198 Gif-sur-Yvette, France; myriam.salameh1@hotmail.com (M.S.); sylvie.riquier@cnrs.fr (S.R.); olivier.guittet@cnrs.fr (O.G.); meng-er.huang@cnrs.fr (M.-E.H.); laurence.vernis@cnrs.fr (L.V.); michel.lepoivre@cnrs.fr (M.L.)

**Keywords:** iron-sulfur protein, CISD2, Fe–S cluster transfer, Fe–S cluster lability, Wolfram syndrome, UV-visible absorption spectroscopy

## Abstract

Human CISD2 and mitoNEET are two NEET proteins anchored in the endoplasmic reticulum and mitochondria membranes respectively, with an Fe–S containing domain stretching out in the cytosol. Their cytosolic domains are close in sequence and structure. In the present study, combining cellular and biochemical approaches, we compared both proteins in order to possibly identify specific roles and mechanisms of action in the cell. We show that both proteins exhibit a high intrinsic stability and a sensitivity of their cluster to oxygen. In contrast, they differ in according to expression profiles in tissues and intracellular half-life. The stability of their Fe–S cluster and its ability to be transferred in vitro are affected differently by pH variations in a physiological and pathological range for cytosolic pH. Finally, we question a possible role for CISD2 in cellular Fe–S cluster trafficking. In conclusion, our work highlights unexpected major differences in the cellular and biochemical features between these two structurally close NEET proteins.

## 1. Introduction

Recently discovered NEET proteins are iron–sulfur (Fe–S) cluster-containing proteins that are present in all the kingdoms of life. Members of this family are characterized by at least one highly conserved 39-amino-acid motif called CDGSH Iron–Sulfur Domain (CISD) harboring a redox-active 2Fe–2S cluster coordinated by an unusual 3Cys–1His quartet instead of the usual 4Cys. In mammals, three NEET proteins have been identified: mitoNEET (also known as CISD1), CISD2 (NAF-1, ERIS, Noxp70 or Miner1) and CISD3 (Miner2 or MiNT) [1,2]. Even if their specific functions are still debated, they are clearly involved in various processes including ageing, autophagy, apoptosis and the regulation of calcium, iron metabolism, Fe–S cluster and reactive oxygen species (ROS) homeostasis. The molecular mechanisms underlying the involvement of NEET proteins in these multiple processes remain poorly understood [2]. 

Several studies reveal that various types of cancers are accompanied by overexpression of NEET proteins [3] and the CISD2 expression level has been proposed as an independent prognostic marker for survival in cancer patients including pancreatic [4] and gastric cancers [5]. More generally, the overexpression of NEET proteins promotes the proliferation of cancer cells, supports tumor growth and metastasis. Conversely, their depletion leads to decreased cancer cell proliferation in breast cancer [6] and neuroblastomas [7]. Recessive mutations in the *CISD2* gene coding for CISD2 are the causative factor for Wolfram syndrome 2 (or WFS2), a rare autosomal recessive neurodegenerative disorder leading to severe neurological disabilities and a drastic decrease in life span [8].

**Figure 1:**
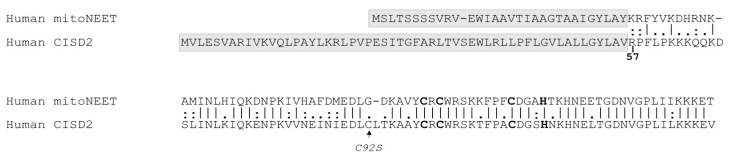
Comparison of the amino acid sequences of human mitoNEET (NP_060934.1, 12.2 kDa) and CISD2 (NP_001008389.1, 15.3 kDa). The following markup lines are indicated: “.”, small positive score; “:”, similarity; “|”, identity. Non-cytosolic parts of the proteins (in-organelle domain and membrane anchors) are marked by grey boxes. Residues involved in cluster coordination are in bold. The C92S point mutation introduced to express the cytosolic domain of CISD2 is indicated by an arrow.

MitoNEET and CISD2 are dimers that assemble one cluster per protomer whereas CISD3 is monomeric and assembles two clusters. While the latter resides inside the mitochondrial matrix, mitoNEET and CISD2 are respectively bound to the mitochondrial and endoplasmic reticulum (ER) membranes by a specific N-terminal anchor with the remaining parts of the protein, including the Fe–S cluster domain, laying into the cytosol [2]. Their cytosolic domains share 65% identity and 79% similarity (Figure 1) with a similar unique folding that exhibits two distinct domains: the Fe–S cluster binding domain and a β-cap domain [2].

One of the most outstanding biochemical properties of the NEET proteins is their ability to transfer their cluster to acceptor apoproteins (cluster transfer reaction) [9,10]. By combining in vitro and in cellulo approaches, our recent data demonstrated the involvement of the human mitoNEET in a novel Fe–S trafficking pathway to quickly rebuild a cluster in IRP-1/cytosolic aconitase following an oxidative insult [11]. Remarkably, the mitoNEET cluster is extremely stable when reduced, even at acidic pH, and can hardly be lost or transferred [12]. However, it can be reversibly oxidized to [2Fe–2S]^2+^ by hydrogen peroxide (H_2_O_2_) and be further reduced by biological thiols suggesting a redox sensory function of mitoNEET [13,14]. Only an oxidized mitoNEET cluster can be transferred in vitro to physiological cluster acceptors such as the cytosolic aconitase and to model cluster acceptors such as *Escherichia coli* ferredoxin (FDX) [12]. Finally, we found that the rate of mitoNEET cluster transfer is highly pH dependent because a much faster reaction occurs at slightly acidic pHs [15]. All these results led us to propose that mitoNEET was acting as a redox switch protein with a pH-dependent cluster transfer activity controlled by the redox state of its Fe–S cluster [15,16]. 

CISD2 has been much less characterized than mitoNEET at the biochemical level and the transfer of CISD2 cluster to acceptor proteins was only shown in vitro using hyperthermophilic ferredoxin [10] and human CIAPIN1 [17]. No CISD2 cluster recipient has been formally identified in cellulo to date. Since human NEET proteins are playing important roles in multiple essential biological processes and in various human diseases, there is an urgent need for a better knowledge, at both biochemical and cellular levels, of each NEET member. Combining cellular and biochemical approaches, the present study focused on human CISD2 and aimed to better delineate what distinguishes it from its closest homolog mitoNEET at the molecular level. We found out that mitoNEET and CISD2 show different expression profiles in mouse tissues. Moreover, CISD2 is highly stable in cells and little sensitive to iron chelator treatments, known otherwise to lead to rapid mitoNEET decay. In vitro, the decreased stability of oxidized CISD2 and mitoNEET clusters in the presence of oxygen (aerobiosis) are similar. In contrast, at acidic pHs, the CISD2 Fe–S cluster is much more stable and is transferred more slowly than the mitoNEET cluster. As a consequence, CISD2 is a poor cluster donor compared to mitoNEET. This work reveals unexpected major differences between human CISD2 and mitoNEET, two structurally close NEET proteins.

## 2. Materials and Methods

### 2.1. Protein Sequence Alignment

Pairwise sequence alignment of human mitoNEET and CISD2 was performed with the EMBOSS Water Website (www.ebi.uk), that uses the Smith–Waterman algorithm to calculate the local alignment of two sequences. The protein comparison is presented with the “pair” output format. A space is introduced for a mismatch or a gap. 

### 2.2. Cell Culture and Treatment

Human epithelial carcinoma (HeLa) cells were cultured in Dulbecco’s modified Eagle medium (DMEM, Sigma–Aldrich, Saint Louis, MI, USA) containing 4.5 g/L glucose, 1 mM stable L-glutamine and supplemented with 1% penicillin–streptomycin and 10% fetal bovine serum (Lonza, Bâle, Switzerland) under 5% CO_2_ and humidified atmosphere. Cycloheximide (CHX, 10 and 50 µM) and desferrioxamine (DFO, 50 µM) were from Sigma–Aldrich. Salicylaldehyde isonicotinoyl hydrazone (SIH, 50 µM) was a kind gift from P. Ponka (McGill University, Montreal, QC, Canada). 

### 2.3. Preparation of Cell Extracts and Immunoblot

Total protein extracts from cultured HeLa cells were obtained by harvesting cells in Laemmli buffer (0.06 M Tris HCl pH 6.8, 10% glycerol, 2% SDS, protease inhibitors (Calbiochem, San Diego, CA, USA)). Protein concentrations were determined using the BCA method. Equal amounts of proteins were separated by SDS–PAGE and transferred on 0.45 µm PVDF membranes. The primary antibodies used were: anti-β-actin (Sigma–Aldrich #A5441), -CISD2 (Proteintech #13318-1-AP), -prohibitin (Novusbio #NBP2-37563), -mitoNEET (designed by Eurogentec). Secondary antibodies were anti-mouse and anti-rabbit fluorescent IRDye 800CW (LI-COR) and membranes were scanned with an Odyssey^®^ Imaging System (LI-COR, Lincoln, NE, USA). 

### 2.4. siRNA Transfections

HeLa cells were seeded at 3.5 × 10^5^ cells percm^2^, incubated overnight and transfected with siRNA duplexes using INTERFERin^TM^ (Polyplus Transfection, Illkirch, France) according to the manufacturer’s recommendations (Life Technologies^®,^ Calsbad, CA, USA). For incubations with siRNA longer than three days, cells were re-transfected. The siRNA duplexes were from Life Technologies^®^: *mitoNEET* (s31650), *cisd2* (s54620), negative control (4390843) and used at a final concentration of 10 nM. 

### 2.5. Protein Expression Levels in Mouse Tissues

Samples were prepared as previously described (1). Of each tissue, 30–100 mg was sonicated in 1 mL of Buffer A (50 mM NaCl, 25 mM Tris HCl pH 7.5, 2 mM ethylenediaminetetraacetic acid (EDTA), 0.5% Triton X-100) with cocktail protease inhibitors (Sigma–Aldrich) on ice for 15 s and then centrifuge at 14,000× *g* at 4 °C for 15 min. Protein content of supernatant was determined using the BCA protein assay. Ten μg of protein extracts were loaded on three 16% reducing polyacrylamide SDS–PAGE gels. Two gels were transferred on PVDF membranes for immunoblotting while the third one was stained using the SimplyBlue SafeStain kit (ThermoFisher Scientific, Waltham, MA, USA).

### 2.6. Purification of Holo-CISD2_57–135_

A construct missing the 56 N-terminal amino acids of *Homo sapiens* (*Hs*) CISD2 (NP_001008389.1) was expressed using pET28b (GenScript). The open reading frame coding for CISD2_57–135_ was cloned between *Nde*I and *Bam*HI sites. To reduce protein aggregation, a C92S mutation was introduced (2). The *Hs* CISD2_57–135_-C92S form will be referred to as CISD2_s_ in this manuscript for ease of reading. The purified protein contains a thrombin cleavable N-terminal His-tag sequence (MGSSHHHHHHSSGLVPRGSHM), which was added to the N-terminal end of the protein. After thrombin cleavage, extra GSHM residues remain at the N-terminal end of CISD2_s_. The protein was expressed in BL21(DE3) cells cultured in LB media. When the culture reached an OD_600nm_ of 0.6, 500 μM FeCl_3_ was added and the temperature was decreased to 20 °C. After 20 min incubation, protein expression was induced at 20 °C by addition of 0.2 mM isopropyl β-D-1-thiogalactopyranoside (IPTG) for 24 h. Cells were harvested for purification steps performed at room temperature and aerobic conditions. Purification buffers were 0.45 µm filtered and degassed. Cell pellets from a 2 L-culture were resuspended in 100 mL of Lysis buffer (20 mM Tris HCl pH 8 500 mM NaCl, 30 mM imidazole) containing antiprotease cocktail (Sigma Fast, Sigma–Aldrich). Cells were lysed using a cell disruption system (Constant Systems Ltd., Daventry, UK). After centrifugation at 30,000 rpm for 1 h, the cleared lysate was applied on an Ni–NTA column using an Äkta FPLC (GE Healthcare, Chicago, IL, USA) and the column washed with 20 mL of Lysis buffer. Cleavage of the His-tag was performed using 100 NIH U of human thrombin (Sigma–Aldrich) in 25 mM Tris HCl pH 8, 500 mM NaCl, 2.5 mM CaCl_2_ for one night at 4 °C. The cleaved protein was eluted using a Lysis buffer. Colored fractions were concentrated to 5 mL using an Amicon Ultra 15 mL 3K (Sigma-Aldrich) concentrator. Then, proteins were loaded on a Superdex 75 HiLoad 16/60 size exclusion column (GE Healthcare) equilibrated with buffer A (25 mM Tris HCl pH 8, 100 mM NaCl). Eluted fractions were analyzed on a 16%, SDS–PAGE gel, pooled and concentrated. Finally, the proteins were loaded on a HiTrap CaptoSP ImpRes 1 mL (GE Healthcare) equilibrated with buffer B (20 mM Tris HCl pH 8) and eluted using a 40 mL linear gradient between buffer B and buffer C (20 mM Tris HCl pH 8, 1 M NaCl). Fractions were pooled, concentrated, aliquoted and stored at −80 °C. Protein concentration was measured using the Bradford assay (Bradford protein Assay, Biorad, Hercules, CA, USA) with bovine serum albumin (BSA) as standard. Protein purity was assessed to be >99% using SDS–PAGE and immunoblot using anti-His-tag (ThermoFisher #MA1-21315) and anti-CISD2 antibodies. For holo-CISD2, the optical A_280nm_/ A_458nm_ ratio was near 2.4. Apo-CISD2 was obtained by loss of the cluster of holo-CISD2 at 37 °C under aerobic condition in Bis–Tris buffer at pH 6.2.

### 2.7. Purification of mitoNEET and Preparation of apo-FDX 

Human mitoNEET_44–108_ was purified as previously described (3). *E. coli* FDX was expressed from the pET21b plasmid (a gift from Dr S. Ollagnier de Choudens, Grenoble, France) and purified as holo-FDX as described previously (1). Protein purity was assessed to be >99% using SDS–PAGE. Apo-FDX was prepared by heat cluster disassembly of purified holo-FDX in the presence of 10 mM dithiothreitol (DTT) and 10 mM EDTA followed by purification on a NAP-5 column (GE Healthcare) equilibrated with 50 mM Tris HCl pH 7, 100 mM NaCl. Protein concentrations were measured using the Bradford assay with BSA as standard.

### 2.8. Circular Dichroism

CD spectra were recorded with a Jasco (Lisse, France) J-810 spectropolarimeter at 25 °C using 20 µM protein solutions in quartz cuvettes with a pathway of 0.1 cm for spectra between 190 and 300 nm and 0.2 cm for spectra between 300 and 700 nm. For each spectrum, 25 accumulations were made. The spectrum of the buffer was recorded with 15 accumulations and subtracted to sample spectra, which was carried out in 10 mM Tris HCl pH 8 for holoproteins. Apoproteins were prepared by incubation overnight at 37 °C under aerobic conditions after buffer exchange to 10 mM Bis–Tris pH 6.2. CD scans from 300 to 600 nm were collected to analyze signature cluster-bound protein peaks at a scan rate of 50 nm/min. Data were processed by use of JASCO Spectra Manager II Analysis. Secondary structure composition was evaluated from the CD spectra using online Bestsel algorithm. 

### 2.9. In Vitro CISD2 Cluster Loss and Transfer Reactions

Reaction buffers were all composed of 100 mM NaCl and 50 mM Bis–Tris (pH 5.8, 6.2 and 6.7) or Tris HCl (for pH higher than 7). For cluster transfer reaction, apo-FDX was pre-incubated with 5 mM DTT for 30 min at room temperature under anaerobic conditions to ensure cysteine reduction. Cluster loss and transfer reactions were followed by UV-visible absorption or migration of aliquots taken at defined times (1 mM DTT was added to the samples before migration) on a 16% native PAGE colored with colloidal Coomassie staining as described in (1).

UV-visible absorption spectra were recorded between 240 and 900 nm with a Cary 100 (Agilent, Santa Clara, CA, USA) spectrophotometer equipped with a temperature control apparatus set to the desired temperature. For spectra taken under anaerobic conditions including the study of the effect of H_2_O_2_ on the stability of oxidized CISD2 cluster, the cuvette was prepared in a glove box (Jacomex, Dagneux, France) and closed with a septum. The 240–900 nm absorption spectra of the reaction mixture were recorded over time and corrected for baseline variations at 900 nm. For the cluster loss reaction, we paid attention to changes in absorbance at 460 nm, which is characteristic of oxidized CISD2 cluster. For cluster transfer reactions, we paid particular attention to changes in absorbance at 460 nm and 415 nm, which are characteristic of oxidized holo-CISD2 and holo-FDX, respectively. At time t, the extent of the loss of oxidized CISD2 cluster was determined using R(t) = A_460_, whereas the extent of the cluster transfer was determined using the ratio R(t) = A_415_/A_460_. As previously described [18], reaction progress at time t was estimated as (R(t)−R_initial_)/(R_final_−R_initial_) with R_initial_, the initial R value at time 0 and R_final_ the R value at the time necessary for reaction completion. If the transfer of Fe–S centers is not total, a theoretical value of R_final_ of 1.1 (ratio corresponding to oxidized holo-FDX) as theoretical maximum ratio was used.

## 3. Results

### 3.1. Tissue-Specific Expression of CISD2

**Figure 2:**
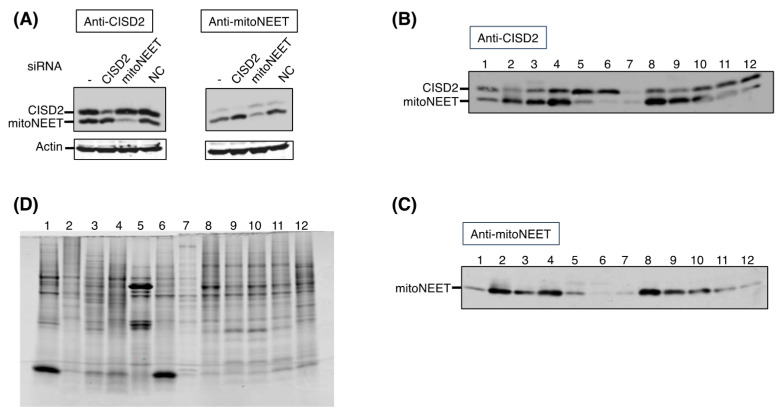
Protein levels of CISD2 and mitoNEET are tissue-specific. (**A**) CISD2 antibody recognized both CISD2 and mitoNEET when mitoNEET antibody was more specific. Cell extracts from untreated HeLa cells (-), or HeLa cells treated for 6 days with CISD2-, mitoNEET- or scramble siRNA (Negative Control, NC) were analyzed on a 16% SDS–PAGE gel by immunoblot using anti-CISD2 antibodies (left panel) and anti-mitoNEET antibodies (right panel). Actin was used as a loading control. (**B**–**D**) Analysis of CISD2 and mitoNEET protein levels in various mouse tissues. 10 µg of protein extracts were analyzed on 16% SDS–PAGE gels. (1) lung, (2) heart, (3) liver, (4) kidney, (5) pancreas, (6) spleen, (7) skeletal muscle, (8) brain, (9) spinal cord, (10) brain stem, (11) cerebellum, (12) testis. (**B**,**C**) Immunodetection of CISD2 and mitoNEET in different tissues of mouse using anti-CISD2 (**B**) and anti-mitoNEET (**C**) antibodies. (**D**) SDS–PAGE gel stained with Simply Blue.

We first checked the specificity of the antibodies and found that the CISD2 antibody recognizes both mitoNEET and CISD2, while the mitoNEET antibody is more specific to mitoNEET (Figure 2A). We then analyzed the levels of CISD2 in protein extracts from various mouse tissues by immunoblotting. Neither of these two proteins is ubiquitous and each has quite specific tissue expression pattern. CISD2 is well expressed in the pancreas, spleen and testis, while mitoNEET is more specifically expressed in the kidney, liver, heart and brain (Figure 2B–D) [18].

### 3.2. CISD2 Is a Long-Lived Protein in Cells

**Figure 3:**
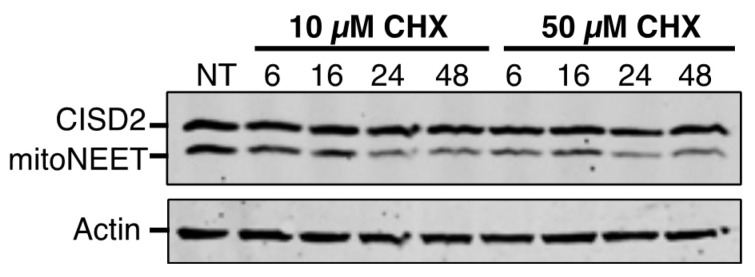
CISD2 is a long-lived protein compared to mitoNEET. HeLa cells were treated with either 10 or 50 µM cycloheximide (CHX) for the specified times. Untreated cells are noted NT. Cell extracts (20 µg) were analyzed on a 16% SDS–PAGE gel by immunoblotting using anti-CISD2 antibody. Actin was used as a loading control.

Using cycloheximide (CHX), we investigated the time-dependent decay of CISD2 in HeLa cells. Over a 48 h treatment with 10 or 50 µM of CHX (Figure 3), the protein level of CISD2 did not decrease significantly even after 48 h, while mitoNEET was much more unstable with a half-life of less than 6 h. CISD2 is therefore a long-lived protein compared to mitoNEET.

### 3.3. Intracellular CISD2 Stability Is Not Senstive to Iron Chelator Treatments

**Figure 4:**
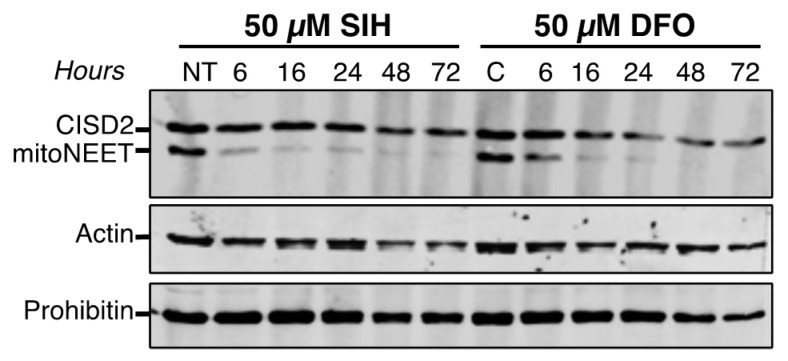
Unlike mitoNEET, CISD2 protein level is moderately affected by iron chelator treatments. HeLa cells were treated with either SIH or DFO for the indicated times. Untreated cells are noted NT. MitoNEET and CISD2 protein levels were determined by immunoblotting using anti-CISD2 antibodies. Actin (cytosolic protein) and prohibitin (mitochondrial inner membrane-localized protein) were used as protein loading controls. The experiment was performed twice; one representative experiment is shown.

We found previously that mitoNEET is degraded by the proteasome when cells are treated with the iron chelators salicylaldehyde isonicotinoyl hydrazone (SIH) or desferrioxamine (DFO) [11]. We similarly treated HeLa cells with 50 µM of either SIH or DFO for up to 72 h and analyzed CISD2 and mitoNEET protein expression (Figure 4). Of note, longer chelator treatments led to an extensive decrease of protein levels of prohibitin (mitochondrial inner membrane-localized protein) and actin (cytosolic protein) (data not shown) indicating a general degradation of the cellular components. CISD2 protein levels over 48 h decreased by only roughly 20% and 50% in the presence of SIH and DFO, respectively, while mitoNEET was barely detectable after 6 h with SIH and 16 h with DFO. Thus, CISD2 protein stability is not significantly affected by iron-chelating agents known to lead to substantial mitoNEET degradation. 

### 3.4. The Fe–S Cluster Is Required for a Global Folding of CISD2

**Figure 5:**
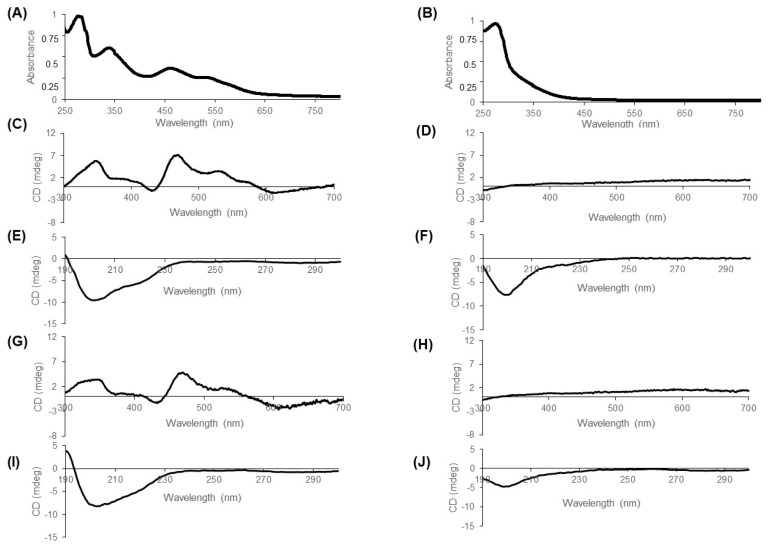
The CISD2 Fe–S cluster is required for global protein folding. Purified CISD2_s_ (20 µM) was analyzed by UV-visible absorbance spectroscopy (**A**,**B**) and circular dichroism (**C**,**F**) and compared to CD spectra for mitoNEET (panels **G**–**J**). (**A**,**B**) UV-visible absorption spectra of holo-CISD2_s_ (**A**) and apo-CISD2_s_ (**B**). Spectra were normalized with an absorbance of 1 at 280 nm. (**C**,**D**,**G**,**H**) CD spectra from 300 to 700 nm of holo-CISD2_s_ (**C**), apo-CISD2_s_ (**D**), holo-mitoNEET (**G**) and apo-mitoNEET (**H**). (**E**,**F**,**I**,**J**) CD spectra from 190 to 300 nm of holo-CISD2_s_ (**E**), apo-CISD2_s_ (**F**), holo-mitoNEET (**I**) and apo-mitoNEET (**J**).

The difference in intracellular stability between CISD2 and mitoNEET led us to question whether this could be related to differences in the role of the Fe–S cluster in the overall folding of the two proteins. In other words, could the overall structure of CISD2 be maintained even without its Fe–S cluster? The cytosolic domain of CISD2 with the C92S mutation to avoid protein aggregation (CISD2_s_) (2) was expressed in *E. coli* and purified. Comparable to mitoNEET, the purification of the protein in aerobiosis was compatible with the maintenance of the Fe–S cluster. The UV-visible absorption spectrum of oxidized holo-CISD2_s_ (Figure 5A) shows the characteristic NEET protein signature with two peaks around 350 and 460 nm and a shoulder around 550 nm. This specific signature is obviously lost in the apo-CISD2_s_ form (without Fe–S cluster) (Figure 5B). 

Both apo- and holoprotein forms were analyzed by circular dichroism (Figure 5C–J) focusing not only on wavelengths above 300 nm corresponding to the signature of the Fe–S cluster [19] but also on the 190 to 300 nm region characteristic of the secondary structure composition of the protein [20]. Holo-CISD2 exhibits CD signal at wavelengths above 300 nm due to the presence of the cluster (Figure 5C). As expected, the loss of the Fe–S cluster leads to the loss of this signature (compare the spectrum of the holoprotein (Figure 5C) with that of the apoprotein (Figure 5D)). For the holoprotein, the region of the spectrum between 190 and 300 nm (Figure 5E) has a main negative peak around 200 nm and a shoulder towards 210–220 nm. This signature is compatible with the composition in secondary structures of CISD2 [21]. Remarkably, this spectrum is clearly flattened in the apo-CISD2, indicating that loss of the cluster leads to major modifications in the secondary structure composition of the protein including loss of α-helices (Figure 5F). Similar observations were made comparing holo- and apo-mitoNEET (Figure 5G–J). As we showed previously for mitoNEET using Nuclear Magnetic Resonance [11], the Fe–S cluster of CISD2_s_ is involved in the maintenance of the secondary structures of the protein that are crucial to its overall folding. Thus, the Fe–S cluster of CISD2 plays a major role in the folding of the protein as for mitoNEET.

### 3.5. Stability of Oxidized CISD2 Cluster Is Moderately pH-Dependent

**Figure 6:**
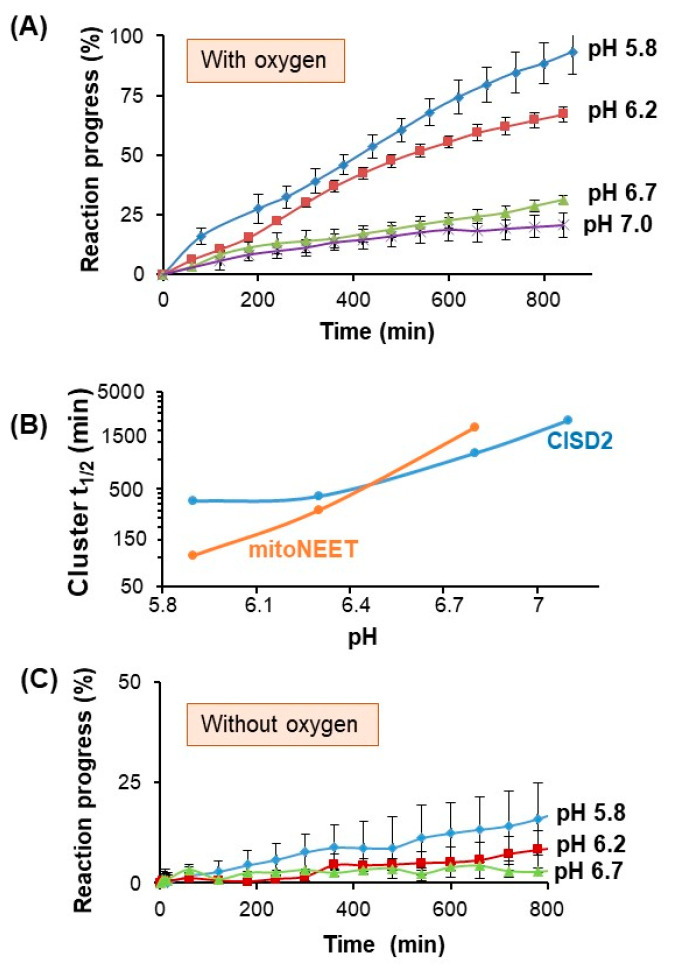
Aerobic stability of oxidized CISD2 cluster is less pH-sensitive than that of mitoNEET. (**A**) pH dependency of CISD2_s_ cluster under aerobic conditions. Oxidized CISD2_s_ cluster loss (20 µM protein) was studied at 25 °C under aerobic conditions and followed by UV-visible absorption spectroscopy. The pH of the reaction (50 mM Tris HCl /Bis–Tris 100 mM NaCl) was 5.8 (blue), 6.2 (red), 6.7 (green), and 7 (violet), respectively. The reaction progress for each type of reaction was calculated as previously described [18]. The graphs are the average curves with error bars from at least three independent experiments. (**B**) Comparison of the pH dependency of the aerobic stability of CISD2_s_ and mitoNEET clusters. Half-life of the oxidized cluster of either CISD2_s_ (blue) or mitoNEET (orange) at 25 °C under aerobic conditions are calculated from the UV-visible absorption experiments presented in panel A (CISD2_s_) and in [15] (mitoNEET). The graph is represented with a log_10_ scale for the *y*-axis. (**C**) Oxidized CISD2_s_ cluster is highly stable under anaerobic conditions. Experiments were performed similarly to panel A but in the absence of oxygen. The graphs are the average curves with error bars from at least three independent experiments.

Could the differences observed in-cellulo between mitoNEET and CISD2 stability (Figure 3 and Figure 4) be related to their respective Fe–S cluster stability? We have previously shown that the presence of O_2_ is a major determinant of mitoNEET cluster stability in vitro with oxidized mitoNEET being less stable under aerobic conditions than in the absence of O_2_ [12]. Moreover, under aerobic conditions, the oxidized cluster stability is highly dependent on pH with a decrease in cluster stability at more acidic pH [15]. Of note, stability of the mitoNEET Fe–S cluster is not decreased at acidic pH under anaerobic conditions, indicating that acidic pH does not lead to a decrease of its intrinsic stability but to an increase of its reactivity with oxygen that ultimately leads to its disassembly. We analyzed the stability of the oxidized CISD2_s_ cluster under aerobic conditions using UV-visible absorption spectroscopy [18]. In resting cells, cytosolic pH is dynamically regulated to near neutral values. Interestingly, at pH 7 and 25 °C, the cluster is relatively stable with only 20% loss in 900 min (Figure 6A). 

Then, we explored the effects of pH acidification, which was observed in cellular events such as early apoptotic steps in which cytosolic pH can drop to 6.3, during myocardial ischemia, and inflammation and infection processes. When the pH gets more acidic, the stability of the oxidized cluster of CID2_s_ decreases under aerobic conditions as previously observed for mitoNEET [15]. However, the comparison between these two proteins shows that the stability of the CISD2_s_ cluster is less dependent on pH than the mitoNEET cluster is (Figure 6B). Around neutral pH, the mitoNEET cluster is the more stable while at an acidic pH the reverse was observed. In the absence of oxygen (anaerobic conditions) and as previously observed with oxidized mitoNEET, the oxidized CISD2_s_ cluster was highly stable even at acidic pHs with a low pH dependency (Figure 6C) [15]. 

### 3.6. CISD2 Is Sensitive to Hydrogen Peroxide Only at Low pH

**Figure 7:**
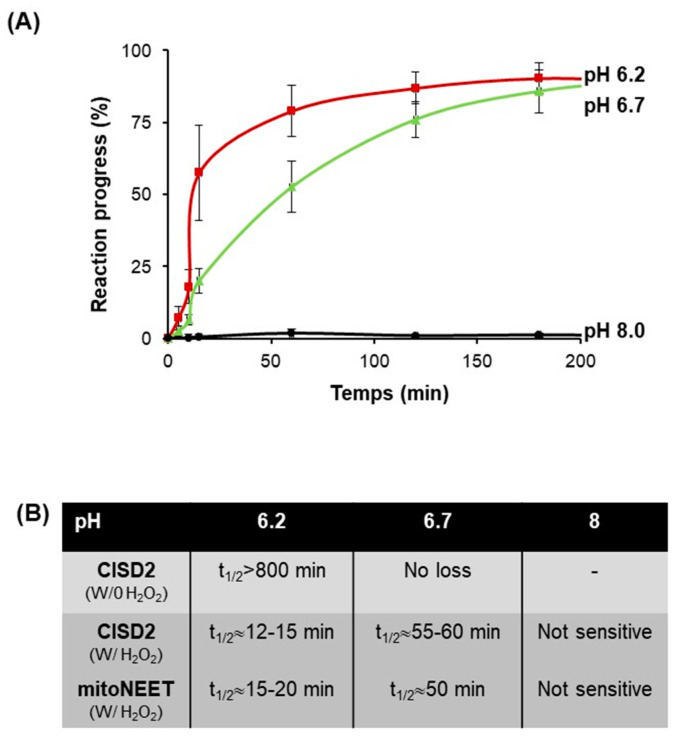
CISD2 is highly resistant to H_2_O_2_ in vitro at neutral pH. (**A**) Cluster loss reactions with 20 µM protein and 250 µM H_2_O_2_ were performed at 25 °C under anaerobic conditions and followed by UV-visible absorption spectroscopy. The reaction buffer (50 mM Tris HCl or Bis–Tris with 100 mM NaCl) was at pH 6.2 (red), 6.7 (green) and 8 (black), respectively. The reaction progress was calculated as previously described. Each experiment was performed at least twice. (**B**) Comparison of the t_1/2_ of the cluster of CISD2 and mitoNEET in the presence of 250 µM H_2_O_2_ at different pHs as described in panel **A**.

We showed previously that the mitoNEET cluster is highly resistant to H_2_O_2_ treatment in vitro compared to other known Fe–S proteins [15]. We investigated the effect of H_2_O_2_ on CISD2_s_ cluster disassembly in vitro under anaerobic conditions, for which CISD2_s_ was stable for hours at 25 °C (Figure 6C). To be consistent with previous studies [22], we first explored the effect at pH 8. At this pH, the addition of 250 µM H_2_O_2_, a high concentration compared to intracellular physiological H_2_O_2_ level, did not significantly destabilize the cluster. However, cluster degradation was readily observed at a pH below 7 and increased when the pH became more acidic (Figure 7A). These results are very similar to those we previously obtained with mitoNEET [15] (Figure 7B). 

### 3.7. CISD2 Is a Weak Fe–S Cluster Donor

**Figure 8:**
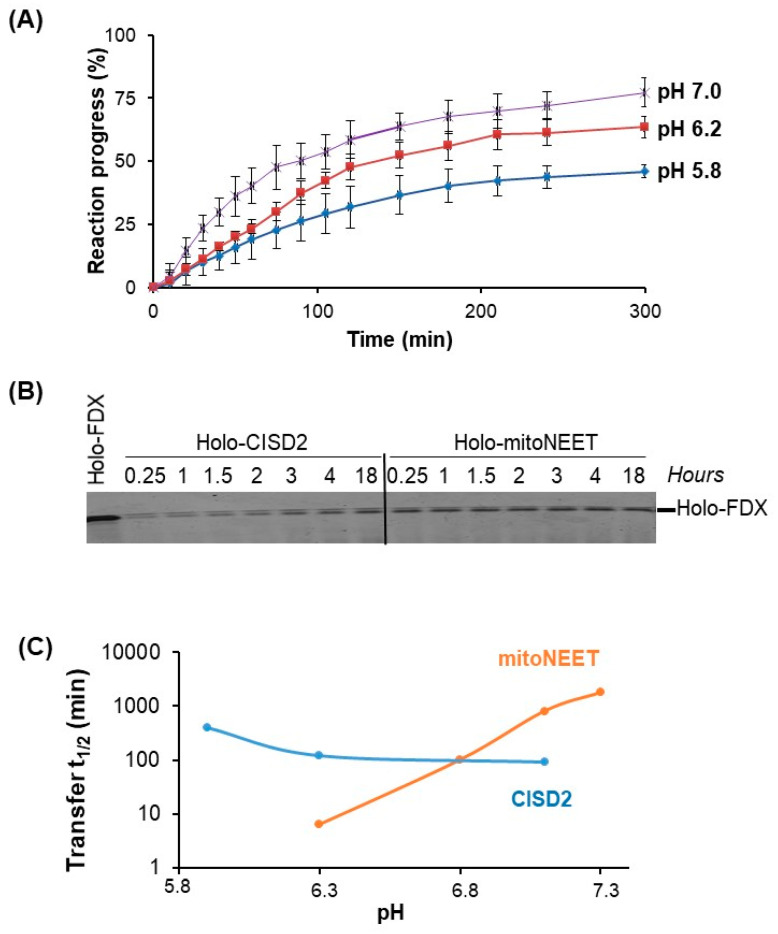
CISD2 cluster transfer reaction is slow with much less pH dependency. CISD2_s_ cluster transfer to apo-FDX (each protein is at 20 µM) was performed at 25 °C under aerobic conditions and followed by either UV-visible absorption spectroscopy (**A**,**C**) or migration on a 16% reducing native gel (**B**). The reaction buffer was 50 mM Tris HCl or Bis–Tris with 100 mM NaCl at various pHs. (**A**) CISD2_s_ cluster transfer as a function of pH was followed by UV-visible absorption spectroscopy. Corresponding pH was 5.8 (blue), 6.2 (red), and 7 (violet), respectively. Reaction progress was calculated as previously described [18]. Each experiment was performed at least three times and the graph is the average curve with errors bars. (**B**) Time course of the formation of holo-FDX using holo-mitoNEET or holo-CISD2_s_ as cluster donor at pH 5.8 analyzed on a reducing 16% native gel. **(C)** Effect of pH on the time necessary to transfer 50% of the cluster from holo-CISD2_s_ or holo-mitoNEET to apo-FDX as a cluster acceptor (t_1/2_), which was calculated from cluster transfer reactions followed by UV-visible absorption spectroscopy presented in panel A (CISD2_s_) and in [15] (mitoNEET). The graph is represented with a log_10_ scale for the *y*-axis.

It was previously shown by others that CISD2 is able to transfer its Fe–S cluster in vitro to a model apoprotein recipient [23] or to human CIAPIN1 [17]. Using *E. coli* ferredoxin (FDX), we previously demonstrated that only the mitoNEET cluster in an oxidized state can be transferred through a mechanism independent of the presence of O_2_ [12]. The reaction speed is highly sensitive to pH with a very fast cluster transfer at slightly acidic pH [15]. Thus, we investigated the ability of CISD2 to transfer its oxidized cluster to apo-FDX in vitro at different pHs using UV-visible absorption spectroscopy. The pH did not drastically affect the speed of the reaction but showed a slightly faster reaction at neutral pH compared to acidic situations (Figure 8A). 

Moreover, none of the reactions went to completion, meaning that some holo-CISD2_s_ still remained even after 900 min (data not shown). Detection of the reaction products on a native polyacrylamide gel (Figure 8B) confirmed that formation of holo-FDX from apo-FDX was fast by using holo-mitoNEET as a source of cluster at pH 5.8 because full cluster transfer to apo-FDX occurs in less than 15 min. The same reaction is much slower using holo-CISD2_s_ (Figure 8B). Thus, holo-CISD2_s_ is a poor cluster donor in vitro compared to holo-mitoNEET at slightly acidic pHs. It should be noted that at neutral pH CISD2 transfers its cluster less slowly than mitoNEET, although the transfer rate is very slow in both cases.

## 4. Discussion

CISD2 and mitoNEET are two NEET proteins anchored to organelle membranes. While CISD2 is anchored at the ER membranes at the contact site with mitochondria, mitoNEET is localized at the outer mitochondrial membranes [2]. However, without considering this difference in anchoring domain and resulting subcellular localization, mitoNEET and CISD2 appear to be very similar. Indeed, their cytosolic domains share high level of sequence identity and structural similarity [2]. In the present study, we compared different characteristics of these two proteins to gain a better understanding of their specificity and mechanisms of action in the cell and highlighted for the first time major differences between these two closely related proteins.

We analyzed the protein level of CISD2 and mitoNEET in different mouse tissues (Figure 2). Contradictory results came from two previous RT–PCR studies [24,25] and the CISD2 protein level had been measured only in a very limited number of organs [26]. We found that, despite expression in many mouse tissues, neither CISD2 nor mitoNEET is ubiquitous. Moreover, their expression profile in tissues differs drastically. We speculate that such differences may reflect their distinct biological functions; notably in the pancreas, a high level of CISD2 expression is accompanied by a low level of mitoNEET. Since both proteins are involved in the regulation of mitochondrial calcium stores through distinct mechanisms [27], the relative dependence of pancreatic cells on CISD2 for this essential regulation might help to understand why CISD2 deficiency in WFS2 patients leads to an early dysfunction of this tissue and diabetes. A similar expression profile in the spleen, where CISD2 also predominates over mitoNEET, suggests an as yet unanticipated possible involvement of CISD2 in immune cells. In contrast, mitoNEET is preferentially expressed in the liver, a key organ for the regulation of iron homeostasis. Interestingly, we previously proposed cytosolic aconitase as one physiological acceptor for the Fe–S cluster of mitoNEET [18]. In fact, we previously established that the expression profile of mitoNEET in mouse tissues was similar to that of cytosolic aconitase. The iron-free form of cytosolic aconitase, IRP-1, controls the expression of different proteins involved in iron metabolism. Therefore, it makes sense to observe a strong expression of mitoNEET, deeply implicated in iron homeostasis, in the liver tissue. Remarkably, the expression profile of CISD2 drastically differs from that of cytosolic aconitase (compare Figure 2 from this manuscript to Figure 7 from reference [18]), consistent with the idea that CISD2 is not a physiological Fe–S donor for cytosolic aconitase in vivo.

It was observed that the extinction of CISD2 expression in mouse embryonic fibroblasts induced a very strong overexpression of mitoNEET, thus supporting the hypothesis that mitoNEET and CISD2 might share redundant functions [28]. However, this assumption has been questioned because tissues from CISD2^−/−^ mice did not show higher mitoNEET protein levels [25]. The same is true in human epithelial breast cancer cells, where the extinction of either one did not affect the level of the other protein [6]. In line with these latter findings, we did not detect increased amounts of mitoNEET or CISD2 when knocking down CISD2 or mitoNEET respectively, in HeLa cells. Taken together, our analysis of CISD2 and mitoNEET expression in different mouse tissues, as well as previously published results from our group and others, substantiate the idea that CISD2 and mitoNEET are much more than just redundant backups in important cellular processes. However, it cannot be excluded that in some specific tissues their activities may overlap at least partially and compensatory mechanisms might exist between these two NEET proteins. 

The proteins’ stability and their sensitivity to iron chelation are a second major point of divergence between CISD2 and mitoNEET. The half-life of a protein ranges from 30 min for the least stable to more than 200 h for the most stable [29]. We established that the half-life of CISD2 is longer than 48 h, which is over 8-fold more stable than mitoNEET in unstressed cells. The low turnover of CISD2 probably reflects its greater importance compared to mitoNEET in fundamental cellular processes, such as calcium transfer from the endoplasmic reticulum to the mitochondria, regulation of autophagy and implication in cell proliferation [3]. On the opposite, the relative instability of mitoNEET might help the protein to function in adaptive cellular responses, for instance under oxidative stress or iron deficiency conditions. In fact, our results show a strikingly different behavior of mitoNEET and CISD2 in response to iron deprivation. In the presence of iron chelators, mitoNEET protein levels fall very rapidly while those of CISD2 are only slightly affected. Therefore, the Fe–S cluster of mitoNEET might be its Achilles heel with regard its stability, meaning that proteases might rapidly eliminate apo-mitoNEET. In fact, we have previously shown that iron deprivation caused a degradation of mitoNEET that was partially blocked by the addition of the proteasomal inhibitor MG132, suggesting a role of the proteasome in the regulation of the apo-mitoNEET half-life [11]. The much higher stability of CISD2 under conditions of iron deficiency might be understood as resulting from a stronger binding of the Fe–S cluster to the protein, from a greater stability of the apoprotein in cells, or from both of these possibilities. It is thus tempting to speculate that, in contrast to mitoNEET, the relative insensitivity of the half-life of CISD2 to intracellular iron levels might indicate that CISD2 has, at most, only a minor role in the adaptive response to iron chelation. On the other hand, as iron chelators are able to induce a strong mitophagy response [30], the catabolism of mitochondrial components in a mitophagic process might also contribute to the loss of the mitochondrial mitoNEET following iron deprivation. Because CISD2 is located in the ER compartment and mitoNEET in the mitochondria, the difference in subcellular localization between CISD2 and mitoNEET might also explain the higher stability of CISD2 compared to mitoNEET in response to intracellular iron depletion. 

Our results with iron-deprived cells raised the question whether CISD2 stability in cells may be linked to the stability of its Fe–S cluster. We found that, like mitoNEET, CISD2 stability is significantly higher in anaerobiosis than in the presence of oxygen. Moreover, the oxidized Fe–S cluster has a high intrinsic stability (results under anaerobic conditions) even at acidic pHs, and its sensitivity to oxygen increases as the pH becomes more acidic. However, the stability of the oxidized mitoNEET cluster exhibited a much more pronounced pH dependency compared to CISD2. At pH 7 and above, both clusters were very stable but the mitoNEET cluster was the most stable. At pH 6.4, the Fe–S clusters of both proteins were equally stable in the presence of oxygen. Then, at pH below 6.4, the stability of the mitoNEET cluster dropped while that of CISD2 was only slightly affected. These two Fe–S clusters are similarly coordinated by 3Cys and 1His. However, cluster ligands are in turn stabilized by a complex network of hydrogen bonds with more distant residues that differs slightly between the two proteins [2]. Investigations are underway to identify those residues in the secondary coordination sphere that might be involved in the stabilization of the CISD2 cluster in the presence of oxygen and its low pH dependency. Interestingly, induction of apoptosis in breast cancer cells leads to CISD2 interaction with the inhibitor of apoptosis-stimulating protein of p53 (iASPP) and a structural model of holo-CISD2 in interaction with iASPP has been proposed [31]. Prohibition of this interaction by a iASPP-derived peptide also inhibits apoptosis in these cells. On the basis of our present results, we can consider that the pH–independent stability of CISD2 cluster would prevent CISD2 unfolding and help maintaining the interaction of CISD2 with iASPP, facilitating apoptosis despite cytosol acidification.

We showed that under anaerobic conditions at pH 8, CISD2 and mitoNEET are not affected by the addition of H_2_O_2_ (Figure 7) while under the same conditions the clusters of the bacterial IscU, involved in Fe–S cluster maturation, and of SufB, also involved in Fe–S maturation but under stress conditions, are quickly degraded (t_1/2_ lower than 5 and 10 min, respectively) [22]. The resistance of the CISD2 cluster to H_2_O_2_ compared to other Fe–S proteins suggests that CISD2 functions involving its prosthetic group are likely to be preserved during oxidative stress. However, we noticed that at lower pHs, the CISD2 cluster became more sensitive to H_2_O_2_. Therefore, we cannot exclude that, under specific pathophysiological conditions leading to a strong decrease of the cytosolic pH, CISD2 cluster could be more sensitive to high concentrations of intracellular H_2_O_2_, resulting in either an increased degradation of the cluster or its transfer to a receiving protein.

Finally, we investigated the ability of CISD2 to transfer its Fe–S cluster to a model apo-acceptor protein in vitro. Unlike mitoNEET, we found that the transfer rate was only moderately affected by the pH. Under our experimental conditions, we never obtained a complete transfer even after 300 min of incubation. At pH 7, the transfer rate was about 360 M^−1^·min^−1^ at room temperature, which is of the same order of magnitude as those published for the transfer of CISD2 cluster to an hyperthermophilic ferredoxin [23] or to anamorsin [17] at 37 °C. Comparing these values to those of common Fe–S cluster transfer proteins involved in Fe–S cluster biogenesis such as Grx5, IscU, NfuA, IscA, reveals that the kinetics of the transfer using CISD2 is very slow, noting that the in vitro transfer rate is typically between 1000 and 50,000 M^−1^·min^−1^ with the vast majority greater than 5000 M^−1^·min^−1^ at room temperature [32]. We wondered if the transfer of the Fe–S cluster of CISD2, which was not completed after 5 h of reaction in our conditions, has any physiological significance. We have previously demonstrated that mitoNEET can repair a damaged cluster of cytosolic aconitase in cellulo after oxidative stress [11]. Thus, the transfer of the mitoNEET cluster can occur in cellulo although the transfer of this cluster in vitro was also slow at a pH close to that of the cytosol (pH 7.4), while fast at a pH slightly below 6 [15]. Thus, either the chaperone proteins might facilitate this process at cytosolic near neutral pH or the local pH near mitoNEET in cells might be slightly acidic, at least under certain physiological conditions favorable for the transfer of the cluster. During the induction of apoptosis, for example, the cytosolic pH can drop to around 6.3 [33]. In the case of CISD2, the in vitro cluster transfer is not efficient at any pH suggesting that CISD2 could be only a weak cluster donor in cellulo. However, we cannot exclude the possibility that the slow reaction in vitro is due to an inappropriate and non-physiological acceptor protein (no acceptor protein of the Fe–S cluster of CISD2 has been identified in cellulo to date) or alternatively that this transfer requires the involvement of facilitating chaperone proteins. In conclusion, while they are very similar in sequence and structure, this study revealed unexpected major differences between CISD2 and mitoNEET regarding their expression in tissues and their cellular and biochemical features. Further investigations are required to decipher in detail their respective biological functions.

## Abbreviations

BSA	bovine serum albumin
CHX	cycloheximine
DFO	desferrioxamine DTT, dithiothreitol
EDTA	ethylenediaminetetraacetic acid
FDX	*Escherichia coli* [2Fe-2S] ferredoxin
IPTG	isopropyl β-D-1-thiogalactopyranoside
OMM	outer mitochondrial membrane
SIH	salicylaldehyde isonicotinoyl hydrazine
WT	wild-type
